# Antimicrobial drug resistance and its significance in Medlife Genesys Clinic in Arad

**DOI:** 10.1186/1471-2334-13-S1-P31

**Published:** 2013-12-16

**Authors:** Dana Negru, Angela Cionca, Laura Nicolescu, Raluca Ianota

**Affiliations:** 1 Public Health Department, Arad, Romania; 2 MedLife Genesys Clinic, Arad, Romania

## Background

Antimicrobial drugs have played a decisive role in decreasing illness and death associated with infectious diseases but selective pressure exerted by antimicrobial drug use also has been the major driving force behind the emergence and spread of drug-resistance traits among pathogenic and commensal bacteria.

## Methods

We conducted a retrospective study on isolates recovered from hospital and ambulatory care settings samples during 2012 to assess antimicrobial drug resistance. Our study has limitations resulted in an incomplete or absent patient information regarding prior treatment history, and potential for bias in selecting isolates that were ultimately tested in this study. Isolate sets cannot be considered truly random; we had no data for prior antimicrobial drug exposure, travel, and other epidemiologic information.

## Results

A total of 157 isolates were tested for pathogenity and 124 for susceptibility to 24 antimicrobial drugs. A significant multidrug resistance (≥3 antimicrobial drug classes) was observed for 47 isolates, including *Staphylococcus aureus*, *Escherichia coli* and *Pseudomonas aeruginosa* (p<0.001), in relation with the origin of samples and patients ages, regardless of medical settings. Methicillin-resistant *Staphylococcus aureus* (MRSA) represented only 17.5% of the *Staphylococcus aureus* specimens. In 10% of cases *E coli* isolates were resistant to fluoroquinolones. Only 15% of *Pseudomonas aeruginosa* isolates were resistant to aminoglycosides, carbapenems and ceftazidime. Surgical wounds were significantly involved in detected resistance to antimicrobial isolates, mainly in young patients, aged 25-34 (p<0.0001). There is no significant difference regarding hospital and ambulatory care settings.

## Conclusion

Our study showed that antimicrobial drug resistance ranged from 9.52% to 50%, with 17.5% MRSA. Fluoroquinolone-resistant *E coli* in community urinary tract infections has the same patterns by either primary or tertiary caregivers.

